# Exacerbation of bronchiectasis by *Pseudomonas monteilii*: a case report

**DOI:** 10.1186/s12879-017-2600-9

**Published:** 2017-07-24

**Authors:** Aditi, Malini Shariff, Kiran Beri

**Affiliations:** Department of Microbiology, Vallabhbhai Patel Chest Institute, North Campus, University of Delhi, Delhi, 110007 India

**Keywords:** *Pseudomonas monteilii*, Bronchiectasis, MALDI-TOF MS, RAPD, *Pseudomonas putida*

## Abstract

**Background:**

*Pseudomonas spp* are important opportunistic and nosocomial pathogens. One such species is *Pseudomonas monteilii (P. monteilii)*. It has been described as an environmental contaminant and potential pathogen. We identified this organism as the causative agent of an exacerbation of bronchiectasis and an environmental contaminant in our hospital on two separate occasions.

**Case presentation:**

*P. monteilii* was the cause of an exacerbation of bronchiectasis in a 30-year-old HIV negative male. Patient presented with cough with sputum production and exertional dyspnea. The isolate was recovered from a sputum sample in significant counts and definitively identified by Matrix-Assisted Laser Desorption/Ionisation- Time of Flight Mass Spectrometry (MALDI-TOF MS). He was treated with piperacillin-tazobactam and recovered clinically and microbiologically. Another two isolates of the organism were contaminants from the hospital environment. The three isolates were susceptible to all tested antibiotics. Typing by Random amplification of polymorphic DNA (RAPD) found no clonal relationship between them.

**Conclusions:**

Less common species of *Pseudomonas* need to be identified accurately. This organism is identified by commonly used phenotypic systems as *P. putida* which may have contributed to a lower reported prevalence. *P. monteilii* is a known environmental contaminant and must also be considered as a potential pathogen, particularly in patients with chronic lung disease.

**Electronic supplementary material:**

The online version of this article (doi:10.1186/s12879-017-2600-9) contains supplementary material, which is available to authorized users.

## Background

The genus *Pseudomonas* consists of non-fermenting gram negative bacilli which are opportunistic human pathogens, plant pathogens and environmental contaminants. The common human pathogens in this genus include the type species *Pseudomonas aeruginosa*, and *Pseudomonas putida. Pseudomonas monteilii* is a species closely related to *P. putida*. It was first isolated from clinical samples almost two decades ago [1], but has since been only rarely isolated from clinical specimens [2, 3]. Most isolates are environmental, including resistant strains from the hospital environment [4]. Though it is considered a coloniser and potential pathogen, its status as a human pathogen is unclear.

During surveillance among environmental and clinical specimens for the presence of *P.aeruginosa*, non-lactose fermenting, oxidase positive gram negative bacilli were subjected to PCR for identification. Two primers targeting different genes which have been used to identify *P. aeruginosa* were employed [5, 6]. Two isolates, one clinical and one environmental, tested positive for the O-Antigen acetylase gene but not for 16S rDNA v2 and v8. These isolates were identified by the Vitek 2 Compact system as *P. putida* and as *P. monteilii* by Matrix-Assisted Laser Desorption/Ionisation- Time of Flight Mass Spectrometry (MALDI-TOF MS) using the MALDI BioTyper software version 3.1 (Bruker Daltonik GmbH, Leipzig, Germany). All isolates showed a score value >2 indicating valid species identification as *P monteilii*. On re-identification of *Pseudomonas spp* isolates by MALDI-TOF MS, another environmental isolate presumptively identified as *P. putida* by the Vitek 2 Compact system was found to be *P. monteilii*. These isolates were not temporally or spatially related.

## Case presentation

The clinical isolate was cultured from a sputum sample of a 30-year-old HIV negative male suffering from an exacerbation of bronchiectasis in September 2015. He was referred to our institute for evaluation of cough with sputum production and repeated episodes of sneezing and nasal discharge for the last 15 years and breathlessness for the past one year. His clinical course was characterised by progressive exertional dyspnoea along with wheezing. A week prior to presentation, he experienced low-grade intermittent fever along with chills and rigors, which prompted the referral. Based on his symptomatic and radiological profile, he had received anti-tuberculous therapy for nine months three years back without relief. He was never a smoker with no history of exposure to environmental smoke, biomass fuel smoke or toxic fumes. General physical examination revealed presence of digital clubbing. There was no evidence of pallor, cyanosis or lymphadenopathy. He was afebrile with a respiratory rate of 18 per minute and oxygen saturation of 94% on oxygen @ 2 L/min. On auscultation, vesicular breath sounds were audible bilaterally along with coarse creptations over all areas of the lung. The total leucocyte count was 17.9 × 10^3^ cells/ mm^3^, with neutrophilic predominance. Spirometry was suggestive of severe restriction with no response to bronchodilators. Chest radiograph showed multiple ring like shadows in bilateral lower zones. High resolution computed tomography of the thorax revealed multiple dilated bronchi with classical signet ring sign and string of pearls appearance in bilateral lower lobes and right middle lobe, suggestive of cystic bronchiectasis.

The patient was admitted to the ward and a sputum sample was sent to the aerobic culture laboratory. Empirical treatment with intravenous infusion of piperacillin-tazobactam 4.5 gm QID and oral azithromycin 500 mg OD was started. On direct microscopic examination of sputum, the sample had 15-20 pus cells and 0-5 epithelial cells/ low power field. Plenty of gram negative bacilli were observed under the oil immersion objective. The sample was processed by semi-quantitative method using a calibrated loop after treatment with N-acetyl cysteine. It was cultured on sheep blood agar and MacConkey agar plates. The plates were incubated overnight at 37 ° C in ambient air and 5% CO_2_ for Blood agar plates. More than 10^5^ cfu/ ml non-hemolytic, 2 mm in diameter, greyish, translucent, moist, low convex colonies on Blood agar and non-lactose fermenting colonies in MacConkey agar, were isolated which were identified as *P. monteilii*. Since significant counts of a single type of organism were isolated from a good quality sputum specimen, it was considered pathogenic. Antimicrobial susceptibility for piperacillin, cefepime, ceftazidime, meropenem, ciprofloxacin, levofloxacin, gentamicin and amikacin was tested using Kirby Bauer's disk diffusion method; for colistin E-test was used (MIC of 2 mcg/ml). *Pseudomonas aeruginosa* ATCC 27853 strain was used as control. The organism was found to be susceptible to all tested antimicrobials [7]. Treatment with piperacillin-tazobactam was continued. Surveillance samples - nasal and pharyngeal swabs, urine and stool specimens were collected on day 1 and day 8 of admission as part of a separate study. None of these samples grew pathogenic organisms. The patient’s symptoms settled, total leucocyte count returned to normal (9.4 × 10^3^ cells/ mm^3^), and sputum culture was negative before he was discharged after eight days of hospital stay. A brief summary of his stay in the hospital has been depicted in the timeline (Additional file 1). On discharge, the patient was shifted to oral piperacillin-tazobactam, which was continued for a further 7 days. On follow up, *P. monteilii* has not been cultured from clinical samples from this patient since.

**Fig. 1:**
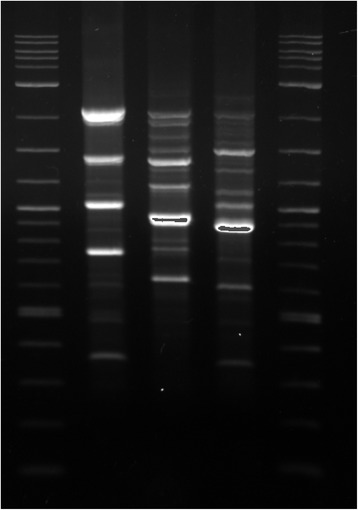
RAPD banding pattern of *Pseudomonas monteilii* isolates. Legend: Lane 1- 2 Log ladder marker, Lane 2- Isolate from sputum specimen, Lane 3-Isolate from bed railing, ICU, Lane 4- Isolate from bedside table, Ward, Lane 5- 2 Log ladder marker

Surveillance cultures from the hospital environment in the month of September 2015 did not grow any *P. monteilii* . The two environmental isolates were cultured one each from a bed railing in the Intensive Care Unit in February 2016, and from a bedside table in the ward in March 2016. Both tested susceptible to all antimicrobials (colistin MIC 1.5 mcg/ml). We did not identify any related clinical isolates in that time period. Though the isolates had identical antibiograms, typing by Random amplification of polymorphic DNA (RAPD) (Fig.1) using the ERIC2 primer found 35–57% homology between the strains in UPGMA generated dice coefficients, indicating no clonal relationship. This was expected since the patient acquired the infection prior to admission and the isolates were recovered months apart. The discriminatory power of this primer is unknown for *P. monteilii*. However, RAPD being affected by differences in factors other than antimicrobial susceptibility, is a more sensitive method for identifying heterogeneity [8].

## Discussion and conclusions

Accurate identification of less common *Pseudomonas spp* is important. In this case, *P. monteilii* was identified by the Vitek 2 Compact system as *P. putida*, which has been previously noted [4]. The prevalence of this organism probably appears to be low due to its absence from the Vitek 2 database. The few studies which have identified it have used either 16S rRNA sequencing (primers 27f and 1525r) or MALDI-TOF MS based identification [2, 9, 10].

*Pseudomonas aeruginosa* is associated with severe disease and rapid decline in lung function in bronchiectasis. Infection once established proves difficult to cure, leading to chronic colonization of the airways. Other species of *Pseudomonas* though less virulent, are also opportunistic pathogens and may cause colonization of the airways. *P. monteilii* is an uncommon but increasingly recognized organism in the hospital environment. In the domestic environment, it has been found to be the most common *Pseudomonas* species isolated and has been cultured most frequently from soil, garbage and drains [10]. It has also been cultured from clinical specimens such as bronchial aspirates, urine, stool, bile and blood though evidence of infection was not collected [1–3]. It was identified as a colonizer in pharyngeal and rectal swabs collected from Syrian refugees, including a meropenem resistant isolate [11]. This organism is also a potential MBL reservoir, with environmental strains having been found to harbour *bla*_VIM-13_ and *bla*_VIM-2_ genes [3–12].

Our observations indicate that *P. monteilii* is also an opportunistic pathogen, probably under-reported due to difficulty in identification. *P. monteilii*, a known environmental contaminant must also be considered as a potential pathogen, particularly in patients with chronic lung disease. It has long been suspected of being potentially pathogenic to humans, and concrete evidence is likely to build with rapid and easy MALDI-TOF MS based identification.

## Additional files

Additional file 1: Stating all the details of the case report including history, diagnosis and treatment. (DOCX 17 kb)

## Abbreviations

MALDI-TOF MS: Matrix-assisted laser desorption/ionisation- time of flight mass spectrometry
MBL: Metallo-β-lactamase
MIC: Minimum inhibitory concentration
RAPD: Random amplification of polymorphic DNA
UPGMA: Unweighted pair group method with arithmetic mean
