# Supplementary material for: Exacerbation of bronchiectasis by Pseudomonas monteilii: a case report
Source: BMC Infect Dis. 2017 Jul 24;17:511. doi: 10.1186/s12879-017-2600-9 (PMC5525215; doi:10.1186/s12879-017-2600-9)
Supplement: Supplementary file 1 — Stating all the details of the case report including history, diagnosis and treatment. (DOCX 17 kb) [file 12879_2017_2600_MOESM1_ESM.docx]

Timeline Picture

Relevant past medical history

H/o having taken Anti-tuberculous therapy three years prior to present episode

2 Sept 2015

2 Sept 2015

10 Sept 2015

**Current Illness**

Cough with sputum production- 15 yrs

Breathlessness- 1 yr

Low grade intermittent fever with chills and rigors-1 week prior to admission

Patient discharged after 8 days of hospitalization. Remained culture negative on follow up.

**Diagnosis**

Bilateral cystic bronchiectasis with acute exacerbation caused by *Pseudomonas monteilii*

**Diagnostic evaluations**

TLC-17.9X 103 cells/mm3, neutrophil predominance

Spirometry: Severe restriction of Airways. CXR: Multiple ring-like shadows lower zone bilateral

CT Thorax: Multiple dilated bronchi with classical signet ring sign and string of pearls bilateral.

Sputum Culture: *P.monteilii*

**Physical Examination**

Digital clubbing, Afebrile, RR18/min

Vesicular breath sounds along with coarse creptations bilaterally

At Discharge

Symptoms settled, TLC returned to normal and sputum culture was negative.

Ongoing Intervention

Piperacillin-Tazobactam continued after culture sensitivity report

Initial Treatment

Empirical: Piperacillin-Tazobactam

Azithromycin
